# Association Between Periodontal Disease and Atherosclerotic Cardiovascular Diseases: Revisited

**DOI:** 10.3389/fcvm.2020.625579

**Published:** 2021-01-15

**Authors:** Faraedon Zardawi, Sarhang Gul, Ali Abdulkareem, Aram Sha, Julian Yates

**Affiliations:** ^1^Periodontics Department, College of Dentistry, University of Sulaimani, Sulaymaniyah, Iraq; ^2^Department of Periodontics, College of Dentistry, University of Baghdad, Baghdad, Iraq; ^3^Division of Dentistry, School of Medical Sciences, University of Manchester, Manchester, United Kingdom

**Keywords:** periodontal therapy, relation, periodontal disease, cardiovascular diseases, atherosclerosis

## Abstract

Atherosclerotic cardiovascular disease (ACVD) is an inflammatory disease of the coronary arteries associated with atheroma formation, which can cause disability and often death. Periodontitis is ranked as the sixth most prevalent disease affecting humans affecting 740 million people worldwide. In the last few decades, researchers have focused on the effect of periodontal disease (PD) on cardiovascular disease. The aim of this review was to investigate the association between these two diseases. PD is a potential risk factor that may initiate the development, maturation, and instability of atheroma in the arteries. Two mechanisms were proposed to explain such association, either periodontal pathogens directly invade bloodstream or indirectly by increasing systemic level of inflammatory mediators. Interestingly, it has been suggested that improvement in the condition of one disease positively impact the condition of the other one. Highlighting the association between these two diseases, the importance of early diagnosis and treatment of PD and its impact on cardiovascular status may be of great value in reducing the complications associated with ACVDs. Further *in vitro* and *in vivo* studies with longer follow up are necessary to confirm the causal relationship between PD and ACVDs.

## Introduction

Periodontal disease (PD) is an inflammatory disease primarily initiated in response to a specific group of bacteria and characterized by a complex host-biofilm interaction (1). According to the World Health Organization, the severe form of periodontitis causes tooth loss in about 5–15% of the population worldwide, and it is considered the sixth most common disease affecting humans (2). Aberrant immune–inflammatory responses determine a patient's susceptibility to developing periodontitis, which may be modified by a range of risk factors (3). The transition from gingivitis to periodontitis initiates when the population and activity of a specific group of periodontal pathogens, predominantly Gram-negative anaerobic bacteria such as *Porphyromonas gingivalis* (*P. ginigvalis*), *Aggregatibacter actinomycetemcomitans* (*A. a), Tannerella forsythia* (*T. forsythia*), *Treponema denticola* (*T. denticola*) and spirochetes, increase in the subgingival biofilm (4). These quantitative and qualitative alterations in the bacterial composition of the biofilm are responsible for disturbing the normal symbiotic relationship between the host and its resident microbiota, leading to an alteration in the hosts immune response. This response can be a “double-edged sword,” in that it is an integral defense mechanism but is also simultaneously responsible for periodontal tissue breakdown proportional with the severity of the disease (5).

The inflammatory response in periodontal tissues is characterized by the local production of various pro-inflammatory mediators and enzymes such as C-reactive protein (CRP), interleukin (IL)-1β, IL-6, tumor necrosis factor (TNF)-α, and matrix metalloproteinases (MMP) (6). Consequently, the rate of periodontal tissue destruction is accelerated with an increase in such mediators. Deep periodontal pockets represent a micro-environment for increased levels of inflammatory cytokines either directly or indirectly. Given the cumulative increase in inflammatory cytokines, and its potential influence on systemic disease processes, this in turn acts as a possible risk factor for several systemic diseases including atherosclerotic cardiovascular disease (ACVD) (7).

ACVDs are a group of disorders affecting the heart and blood vessels, including coronary heart disease, cerebrovascular and other peripheral artery diseases, congestive heart failure, carotid heart disease, and aneurysms. Some ACVDs include two major conditions: ischemic heart disease and cerebrovascular disease which are considered as the first and third cause of death, respectively (7). In Europe, ACVD is responsible for approximately 3.9 million deaths (45% of deaths) annually (8). According to a global survey, there were an estimated 422.7 million subjects with ACVD and 17.9 million deaths due to ACVD in 2015 (9). The pathogenic process causing ACVD is very complex. It is recognized that elevated level of low density lipoprotein cholesterol is the principle element in the pathogenesis of ACVD that change cellular permeability and has impact on arterial walls. Inflammatory cells and cytokines induce plaque formation in the walls of blood vessels, and are also responsible for propagation and rupture of the established plaque along with the resultant thrombotic complications (10).

Mechanisms that have been proposed to explain the link between PD and ACVD include the inflammatory pathways common to both diseases, such as increased levels of white blood cells (WBC), CRP, fibrinogen, intercellular adhesion molecule-1 (ICAM-1), and proinflammatory cytokines (11). Additionally, both diseases share similar risk factors such as smoking, poor oral hygiene, diabetes mellitus (DM), obesity, stress and reduced physical activities. Despite these common features, it is difficult to conclude that periodontitis is a primary causal factor of ACVD, as a result of the complexity in the confounders that correlate PD to ACVD (12).

Although stronger and more suggestive evidence has emerged to highlight a causal relationship between the two pathologies, but it was still insufficient for PD to be classified as a causal risk factor (13). Therefore, further studies are required to provide a more robust, consistent link in order to confirm PD as an independent and potentially adjustable risk factor for ACVD (12). Therefore, the current review attempts to review and update the current evidence and provide further insight into the relationship between PD and ACVD.

## Pathogenesis of Atherosclerotic Cardiovascular Diseases Including the Role of Periodontal Disease

Atherosclerotic disease is a focal thickening of vascular intima residing between the endothelial lining and smooth muscle cell (SMC) layers of blood vessels in response to an immune response (14). Endothelial dysfunction is the earliest change in atherosclerotic formation. The primary etiological factor of atherosclerosis is un known (15). However, other risk factors significantly contribute to the development and progression of this pathology, such as aberrant profile of plasma cholesterol, smoking, hypertension, DM, and increased levels of inflammatory mediators including CRP and cytokines (15).

Atherosclerosis starts with accumulation of low density lipoprotein (LDL) within the intima layer where it is oxidized. This in turn activates increased expression in nearby endothelial cells of cell surface proteins such as ICAM-1, vascular cell adhesion molecule-1 (VCAM-1), and selectins (15). Adhesion of circulating inflammatory cells (monocytes, lymphocytes) to these adhesion molecules is increased by their diapedesis into the inflamed intima site (15). The initial development of the atherosclerotic lesion occurs through differentiation of monocytes to macrophages that scavenge on LDL, thus forming foam cells and subsequently fatty streaks (15, 16). Later, a T-leukocyte induced-cell-mediated immune response with increased level of inflammatory cytokines such as INF-γ, TNF-α, and IL-1β further accelerate atherogenesis (17). T-cell-associated mediators stimulate migration and mitosis of SMC to form a fibrous pseudo-capsule around the lesion (17). Macrophages loaded with lipids undergo apoptosis leading to formation of a necrotic core underneath the fibrous capsule, which renders it susceptible to rupture, thus leading to formation of fatal thrombosis (14).

Cumulative evidence from literature over the last decades have supported the role of PD as an independent risk factor for ACVD (18). The presence of certain periodontal pathogens, Gram-negative anaerobes in particular, in subgingival biofilm has been associated with increased risk of MI; the odds ranging between 2.52 and 2.99 with the presence of *T. forsythia* and *P. gingivalis*, respectively, in comparison to controls (19). The hallmark of periodontitis is elevation in the levels of Gram-negative bacteria that are characterized by their ability to trigger an intense immune response via their mechanism of pathogenicity, such as lipopolysaccharide (LPS) (20). Moreover, some of these bacterial species possess the potential to invade deeper tissues, reaching the circulation and inducing a systemic immune response away from their original habitat (21). Results from several *in vivo* and *in vitro* studies have suggested that periodontal bacteria associated with chronic inflammation may compromise the epithelial-barrier function by epithelial-mesenchymal transition (22–24). Epithelial-mesenchymal transition comprises cellular events starting with loss of polarity, cytoskeletal, and adhesion proteins, ending with loss of epithelial-phenotype and acquisition of mesenchymal-like characteristics (25). This results in loss of epithelial sheet coherence and formation of microulceration; thus, facilitating the penetration of motile periodontal pathogens/virulence factors to the underlying connective tissue and exposed blood vessels. On the other hand, periodontal bacteria can invade host cells as part of their defensive strategy to evade host immune responses (26). This intracellular localization provides not only protection from the body's defensive mechanisms but also a shelter from action of antimicrobials (26). Periodontopathogens such as *P. gingivalis* residing within the cells either stay dormant or multiply by modulating cellular machinery (27). Once multiplied, *P. gingivalis* leaves the epithelial cells via the endocytic recycling pathway to infect other cells or gain access to the circulatory system (28). The trafficking of *P. gingivalis* into endothelial cells is positively influenced by bacterial load, and certain virulent proteins such as gingipains, fimbriae, and hemagglutinin A (29). Further, invasion of gingival epithelial and endothelial cells by *P. gingivalis* could be synergized by *Fusobacterium nucleatum* (30) and *T. forsythia* (31).

Two mechanisms have been proposed to explain how PD influences ACVD. First, a direct mechanism by which periodontal pathogens directly invade endothelial cells. This notion is supported by polymerease chain reaction assays for atherosclerotic plaques (32). Analysis of cardiovascular specimens containing thrombus tissues demonstrated that *Streptococcus mutans* was the most prevalent bacteria (78%), followed by *A. a* (33). Atherosclerotic lesions formed in coronary arteries also exhibited the presence of other bacteria such as *P. gingivalis, Prevotella intermedia*, and *T. forsythia* (34). It is not clear how the presence of bacteria intracellularly influences atherosclerosis but some pathogens, e.g., *P. gingivalis*, could trigger foam cell formation or their persistence within the cells, and thereby provoke a state of secondary inflammation that leads to endothelial dysfunction (35).

Increased systemic levels of inflammatory cytokines due to PD is the second suggested mechanism (indirect pathway). PD stimulates a systemic inflammatory response which results in chronically elevated levels of different cytokines, also related to atherosclerotic vascular disease, such as IL-1β, IL-6, IL-8, TNF-α, and monocyte chemoattractant protein-1. Some can enhance rapid hepatic synthesis and secretion of intravascular plasma proteins such as CRP protein and fibrinogen (36, 37). Additionally, bacterial products such as LPS could enter the circulation and induce a potent immune response. These aforementioned factors could initiate atherosclerosis by their action on endothelial cells, modulating lipid metabolism, and increasing oxidative stress (38). This was supported by results from a previous study that indicated endothelial dysfunction in patients with periodontitis (39).

**Figure 1:**
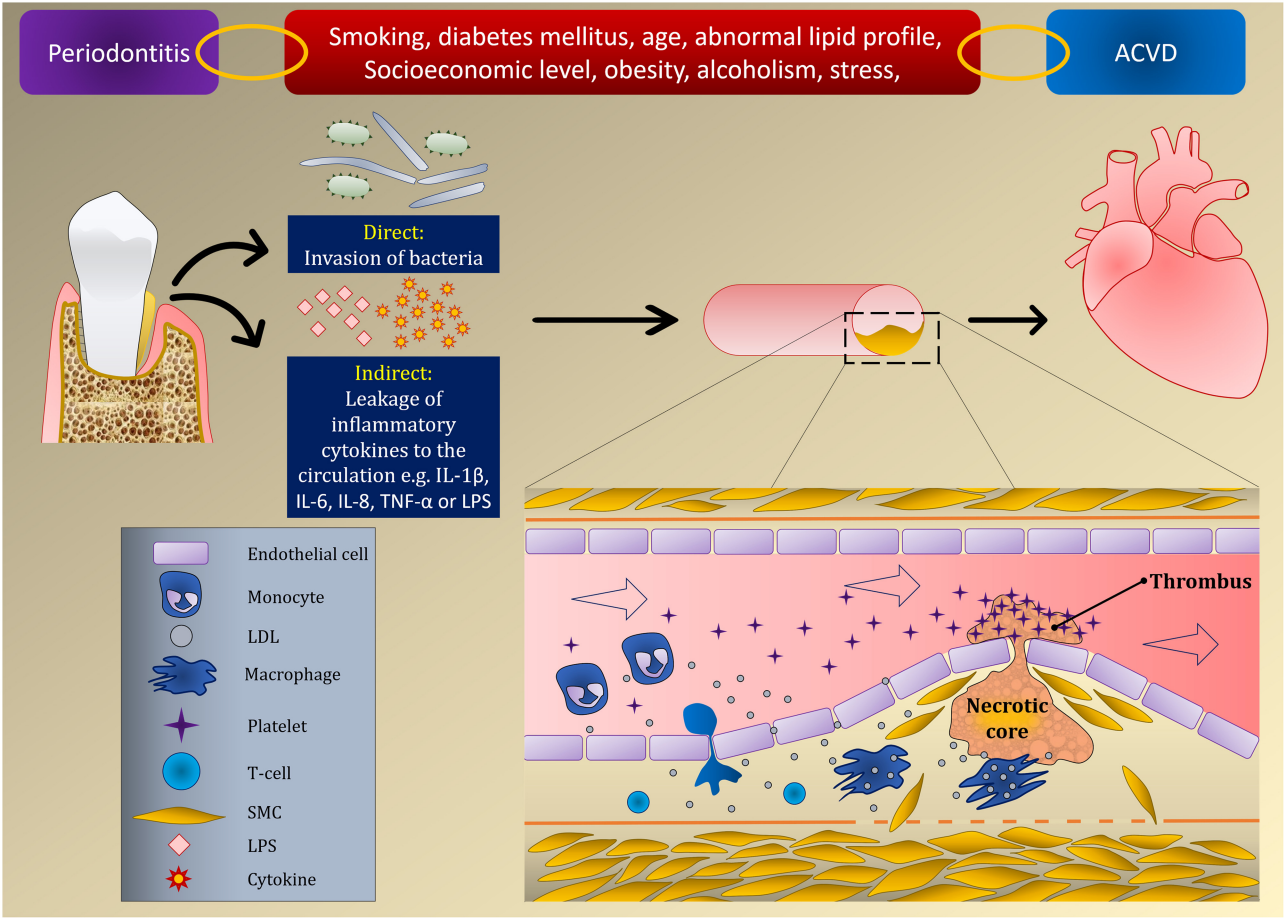
Relationship between PD and ACVD induced by endothelial dysfunction.

Despite robust evidence drawn from many studies linking PD to the initiation and progression of ACVD (which is discussed in section Effects of Periodontal Disease on the Incidence of Atherosclerotic Cardiovascular Diseases), these results require further support to pinpoint the exact pathological mechanism(s) between PD and ACVD. The presence of atherosclerotic diseases and their hypothesized relation with PD are illustrated in Figure 1.

## Confounders Between Periodontal and Atherosclerotic Cardiovascular Diseases

As previously discussed, the available literature has provided ample evidence in relation to the existence of a relationship between PD and ACVD. However, this link is not easily comprehended and it could be further complicated by the presence of other systemic diseases, genetic factors, and lifestyle-related habits. These factors could simultaneously influence the progression of PD and ACVD.

Chronic stress is a response associated with stimulation of a sympathetic nervous system which induces the adrenal glands to increase secretion of adrenalin and cortisol in order to cope with the stress (40). Furthermore, stress activates the hypothalamus-pituitary-adrenal axis, which together with a trigger from the sympathetic nervous system causes upregulation of catecholamines, glucocorticoids and inflammatory cytokines (41). Limited studies are available on the influence of stress on the progression of PD in humans; however, it is well-documented that the systemic level of inflammatory cytokines is significantly increased in response to prolonged stress (41). These cytokines are common to destructive events of PD. Experimental *in vitro*, and *in vivo* animal studies have provided potential mechanisms by which stress can contribute to periodontal tissue breakdown (42). Concomitantly, several types of stress are involved in the development of cardiovascular disease, including oxidative stress, mental stress, hemodynamic stress and social stress. The relationship of stress and ACVD has been thoroughly investigated and the results suggest that individuals suffering from ACVD and under psychological stress are more prone to transient myocardial ischemia, risk of recurrent ACVD and increased mortality (41).

Smoking is a well-recognized risk factor for PD and ACVD. Among toxic products generated during smoking, nicotine is one of the most harmful (43). This substance is responsible for vasoconstriction that compromises delivery of nutrients to the periodontium. In addition, nicotine significantly suppresses cellular/humoral immune responses and causes neutrophil dysfunction (43). Similarly, toxic products produced during burning of tobacco induce the atherogenic mechanism by increasing oxidation of LDL, causing chronic inflammation in the intima layer and subsequent endothelial dysfunction (44, 45). Smoking increase platelet aggregation, blood viscosity and shifts the pro- and antithrombotic balance toward increased coagulation. The contribution of smoking to the pathophysiology of PD and ACVD has been demonstrated by a significant reduction in the strength of association between these two diseases after adjustment of smoking (46). A systematic review showed that 11 out of 15 cross-sectional studies had suggested a modest relationship between ACVD and PD after adjusting for several risk factors including smoking (47).

Diabetes mellitus (DM) is a metabolic disease that adversely affects the body through different mechanisms including periodontal and cardiovascular health. Persistent increase in glucose level is expressed as microvascular changes leading to endothelial cells dysfunction (48), mainly via increased level of TNF-α, interleukins and proteinases that enhances apoptosis of endothelial cells (49). DM-associated complications also affect the bone healing capacity, mineral density, and turn-over rate (50). These DM-associated systemic events significantly increase progression rate of PD. The odds of periodontitis are increased by almost three- to four-fold in diabetic patients compared to healthy controls (51). This is supported by several studies that demonstrated an increase in the prevalence and severity of PD regardless of age, gender, and ethnic group (52). Undoubtedly, DM is a profound risk factor for initiation and progression of periodontitis and ACVD that should be considered when studying the association between these two diseases.

Both periodontitis and ACVD are multifactorial diseases whose development and progression require interaction between several factors, among which is genetic predisposition. A study conducted on twins, utilizing quantitative genetic analyses, showed evidence of an association between ACVD and PD (53). Interestingly, three of the loci among four genes significantly associated with PD, namely ANRIL/CDKN2B-AS1, PLG, and CAMTA1/VAMP3, showed association with ACVD (54). Furthermore, results from a candidate-gene association study also concluded that periodontitis and ACVD are genetically related through at least one susceptibility locus (55). Despite the fact that these studies highlighted a novel shared pathologic pathway between the two conditions, larger scale genetic studies are highly recommended.

Any potential contribution of periodontitis to the pathology of ACVD should be carefully interpreted as many confounding factors could affect both conditions and result in overestimation of this relationship. Thus, adjustment of these risk factors need to be taken into consideration during statistical analysis.

## Biomarkers Shared by Periodontal and Atherosclerotic Cardiovascular Diseases

Endothelial dysfunction is the earliest stage of atherosclerosis and a possible link between PD and ACVD (56). Several studies have linked periodontitis to endothelial dysfunction and this relationship is sustained by several shared biomarkers of periodontitis, ACVD and endothelial dysfunction (47). Despite the potential for these biomarkers to identify the strength of this correlation, they are still not considered as “gold standard” diagnostic markers (47). Upon initiation of periodontitis, expression of inflammatory cytokines markedly increases together with alteration in the lipid profile which could contribute to the development and aggravation of thrombogenesis and thromboembolic events (57). It has been reported that PD is significantly associated with upregulation of biomarkers responsible for endothelial dysfunction and dyslipidemia such as CRP, tissue plasminogen activator (t-PA), and LDL-cholesterol (C), TNF-α (58). Additionally, periodontitis is associated with higher levels of other inflammatory serum biomarkers including von Willebrand factor (vWF), fibrinogen, and endothelial progenitors' cells (58). Interestingly, levels of these serum biomarkers are reduced following periodontal therapy (59, 60).

A systematic review investigated the serum level of a group of mutual biomarkers in order to define the strength of evidence relating PD, CVD, and endothelial dysfunction. The analysis of results indicated that the levels of different inflammatory markers, IL-6 and CRP in particular, were elevated. These outcomes of this systematic review suggested that endothelial dysfunction may be the link between PD and ACVD (61). Furthermore, it was found that ACVD is associated with more severe periodontitis and this was marked by higher serum level of high sensitivity (hs)-CRP (62). Elevated level of hs-CRP due to periodontitis exerts stress additional to the previously existing inflammatory activity of atherosclerotic lesion; consequently, increasing the risk of ACVD (63). Recently, periodontitis was found to be associated with high levels of IL-6, PTX3, and sTWEAK in patients with cerebral small vessel disease, increasing by almost 3 times the likelihood of having this type of ACVD (64). This was supported by results from an *in vivo* study that indicated changes in vascular inflammatory biomarkers, IL-6, PTX3, and sTWEAK, in systemic circulation after injection of LPS from *P. gingivalis* in rats (65).

Indeed, the current literature has provided valuable information about shared biomarkers between PD and ACVD which may offer predictive and diagnostic potential to significantly reduce the risk of developing undesirable cardiac events at earlier stages (Table 1). However, further studies are required in this regard as the exact signaling downstream of ACVD and PD biomarkers has not yet been fully elucidated.

**Table 1:** Studies correlating ACVD and PD biomarkers.

**References**	**Aims**	**Study groups, *N***	**Sample**	**Biomarkers, assays^†^**	**Clinical parameters^¶^**	**Results and conclusions**
Joshipura et al. (58)	Health Professional Follow-up Study to evaluate associations among periodontal disease, tooth loss, and specific biomarkers in blood	Male health professionals (*N =* 18,225)	Blood samples	• CRP measured by an ultra-sensitive immunotechnique • Fibrinogen level by the Clauss method • Factor VII, t-PA, sTNF-R, and vWF antigen concentrations assayed by ELISA.	Self-reported periodontal disease and numbers of natural teeth	• CRP, t-PA, and LDL-C significantly ↑ in subjects with self-reported periodontal disease. • Results suggested that periodontal disease is significantly associated with biomarkers of endothelial dysfunction and dyslipidemia
Gita et al. (66)	• Assessment of total cholesterol, LDL, HDL, and triglycerides in periodontal health, and disease • Assessing associations between elevated lipid profiles and periodontal disease	• Control, patients with healthy periodontium (*N =* 30) • Case, patients with PPD ≥5 mm (*N =* 30)	Venous blood	Lipid profile assessed by homogenous enzymatic calorimetry	Oral hygiene index simplified, PPD, CAL, FI, Mobility index, and OPG for assessing bone level	No association between total cholesterol, LDL, HDL, and triglycerides with periodontal disease
Domingues et al. (67)	Assessing the association of markers of cardiovascular risk with severity of periodontitis	• Control (*N =* 45) • Case (*N =* 45)	Venous blood	• LDL- measured by immunoenzymatic assay • CRP, hs-CRP, HDL-c, assayed by immunoturbidimetry automated methodology • Ratio of hs-CRP/HDL-c	• Control group, < 4 sites with PPD ≥ 4.0 mm and CAL ≥ 3.0 mm • Case group, 30% of sites with PPD ≥ 4.0 mm and CAL ≥ 3.0 mm	Severity of periodontitis was inversely and significantly associated with plasma concentrations of HDL-c
Kalburgi et al. (68)	To investigate the serum CRP level, leukocyte count in periodontitis patients and their association with severity of periodontitis	• Healthy control (*N =* 10) • Periodontitis patients (*N =* 20)	Venous blood	• CRP assayed by turbidimetric immunoassay • Determination of TLC and DLC	• Control group; PPD < 3 mm, CAL < 3 mm • Moderate periodontitis; PPD = 4-6 mm, CAL = 3-4 mm • Severe periodontitis; PPD > 6mm, CAL ≥ 5 mm	• CRP in moderate and severe was significantly higher than control • TLC count and neutrophil in particular were significantly higher in severe than moderate periodontitis • ↑ inflammatory burden during periodontitis may ↑ the risk for cardiovascular events
Ramírez et al. (69)	Evaluation of endothelial function, inflammatory biomarkers and subgingival microbial profile associations in patients with and without periodontal disease	• Control, patients with gingivitis and incipient periodontitis (*N =* 20) • Case, patients with moderate to severe periodontitis (*N =* 20)	• Venous blood • Subgingival plaque samples	• Endothelial function measured by FMD • Bacterial characterization by DNA extraction and PCR • MMP-9, MPO, PAI-1, E-Selectin, ICAM-1, adiponectin, and VCAM-1 measured by multiplexed immunocytometric assay	• Control group, clinically healthy periodontium • Case group, patients with at least 10 sites with PPD ≥5 mm + alveolar bone loss in periapical radiographs	↑ population of red complex group, E-selectin, MPO, and ICAM-1 significantly increased in moderate and severe periodontitis cases, suggesting their susceptibility to develop cardiovascular events
Gupta et al. (70)	Clinical trial aimed to correlate the levels of sCD40 L and MCP-1 in serum and GCF of patients with periodontitis before and after SRP	• Healthy control (*N =* 15) • Severe periodontitis patients (*N =* 30)	• Venous blood • GCF	The sCD40 L and MCP 1 levels were quantified using ELISA	• PI, GI, PPD, CAL • Control: GI <1, PPD < 3 mm, CAL = 0 • Case: patients with two or more inter-proximal sites with CAL ≥ 6 mm, not on the same tooth, and one or more inter-proximal sites with PD ≥ 5 mm	• In periodontitis cases, the sCD40 L levels correlated strongly with MCP-1 levels in both GCF and serum before and 6 w after SRP. • The results highlighted the potential benefits of good oral hygiene level on cardiovascular health
Cotič et al. (71)	To investigate the association between oral health and serum biomarkers among the hemodialysis (HD) patients	Adult patients undergoing maintenance dialysis (*N =* 111)	Blood samples	• CRP level was determined by a chemiluminescent immunometric high sensitivity assay • TnT was measured by an electrochemiluminescence assay • NOx concentration was measured by a colorimetric non enzymatic assay Serum levels of IgA and IgG to *Aa* and *Pg* were determined by ELISA	DMF, API, SBI, and CPITN	Levels of CRP and TnT were higher in edentulous patients, indicating a need for improving dental care to retain the teeth as long as possible
Pedroso et al. (72)	Investigate the concentration of modified (m)LDL level in diabetic type 2 patients with periodontal disease and the effect of periodontal therapy on mLDL and diabetes status over 12 months	• Group 1: diabetic patients with periodontitis (*N =* 24) • Group 2: diabetic patients with gingivitis (*N =* 24)	Blood samples	Glycemia, A1c, total cholesterol, HDL-c, LDL-c, triglycerides, hs-CRP, and oxLDL determined by bicinchoninic acid (BCA) protein assay kit and Z-Scan technique for LDL-c	• PPD, CAL, BOP, PI, and GR • CAL >3 mm in two non-adjacent teeth and the CAL ≥5 mm in 30% or more of the teeth present At least 20 teeth present	• Levels of hs-CRP in Group 1 showed a significant reduction after 12 months • Periodontal treatment improved the LDL-c quality in both groups • Periodontal therapy may help with the control and prevention of hyperglycemia and precursors of cardiovascular diseases
Ameen et al. (73)	• Investigate the levels of the cardiac biomarkers in smoker vs. non-smokers in periodontitis and healthy subjects Assessing the level of cardiac biomarkers with severity of periodontitis	• Control (*N =* 20) • Smoker/periodontitis (*N =* 28) • Non-smoker/periodontitis (*N =* 32)	Venous blood	• AST, ALT, CK, and LDH measured by UV/Vis Spectrophotometer • Tr-I assayed by ELISA	• PI, BI, PPD, and CAL • Diagnosis criteria for periodontitis: Stage II and Stage III (Grade B or C) patients (CAL ≥ 3 mm)	• Tr-I, ALT, AST, LDH, and CK significantly higher in smokers than healthy group • Tr-I, CK, and LDH significantly ↑ in smoker/periodontitis vs. non-smoker/periodontitis • Cardiac biomarkers ↑ during periodontitis and their expression is further aggravated in conjugation with smoking
Boyapati et al. (74)	To compare and correlate the occurrences of periodontitis with serum levels of cardiac-biomarkers in patients with coronary heart disorders	• Patients with coronary artery diseases (*N =* 63) • Control: patients without periodontitis (*N =* 31) • Test: patients with periodontitis (*N =* 32)	Blood samples	LDL, HDL, VLDL, TC, hs-CRP, Troponin T, Troponin I, Myoglobin assessed by chemiluminiscence immunoassay	Periodontitis was defined as: • BOP • CAL≥ 1 site • At least 20 natural teeth • At least two sites with PPD > 3 mm	• Patients in test group exhibited significant and positive correlation between TC, VLDL, hs-CRP, Troponin T and periodontal parameters • Prevention of progression of periodontitis potentially reduces the risk of cardiovascular problems

† *CRP, C-reactive protein; hs-CRP, high sensitive C-reactive protein; HDL-c, high-density lipoproteins; LDL-, electronegative low-density lipoproteins; FMD, Flow-Mediated Dilation; PCR, Polymerase Chain Reaction; MMP-9, matrix metalloproteinase; MPO, myeloperoxidase; PAI-1, Plasminogen activator inhibitor type-1; ICAM-1, Intercellular Adhesion Molecule-1; VCAM-1, Vascular Cell Adhesion Molecule-1; AST, aspartate transaminase; ALT, alanine transaminase; Tr-I, troponin-I; CK, creatinine kinase; LDH, lactate dehydrogenase; ELISA, enzyme-linked immunosorbent assay; sCD40 L, soluble CD40 ligand; MCP-1, monocyte chemoattractant protein-1; TLC, total leukocyte count; DLC, differential leukocyte count; t-PA, tissue plasminogen activator; sTNF-R, soluble tumor necrosis factor receptors; vWF, von Willebrand factor; c, cholesterol; ox, oxidized; TnT, cardiac troponin T; NOx, nitrite/nitrate; Aa, A. actinomycetemcomitans; Pg, P. gingivalis; VLDL, very LDL; TC, total cholesterol*.

¶ *PPD, probing pocket depth; CAL, clinical attachment loss; FI, furcation involvement; OPG, orthopantomogram; PI, plaque index; BI, bleeding index; GCF, gingival crevicular fluid; GI, gingival index; SCR, scaling and root planing; GR, gingival recession; DMF, decayed-missing-filled index; API, approximal plaque index; SBI, sulcus bleeding index; CPITN, Community Periodontal Index of Treatment Need. ↑ mean increase*.

## Effects of Periodontal Disease on the Incidence of Atherosclerotic Cardiovascular Diseases

The joint workshop between the European Federation of Periodontology (EFP) and the American Academy of Periodontology (AAP) in 2012 presented evidence linking PD and ACVD (75). The evidence included the role of periodontopathogenic bacteria in ACVD and clinical (epidemiological and intervention) studies that support the association between these two diseases (76) which will be highlighted in this section.

### Microbiological Studies

Clinically, it is very difficult to find the causative agents of atherosclerosis. Firstly, the endothelial injury usually develops and progresses without symptoms, potentially masking the initiating agent. Secondly, multiple factors can lead to a common inflammatory response such as an atherosclerotic lesion, and these factors could be co-existent, which further complicates identifying the causative factor (as discussed in section Confounders Between Periodontal and Atherosclerotic Cardiovascular Diseases). Additionally, studies relating to interventions performed in this respect have reported mixed results, such as no change, temporary worsening of signs after periodontal treatment or improvement in signs (75, 77). Nevertheless, any reported evidence on the potential role of periodontal pathogens in promoting atherosclerosis has to fulfill the following seven proofs (76).

*Proof 1: Periodontal bacteria can reach systemic vascular tissues* Undoubtedly, many studies have shown that oral bacteria in general and periodontopathogenic in particular can enter the systemic circulation and cause bacteremia (12, 14, 76, 78–80). A previous systematic review has shown that following periodontal procedures the incidence of bacteremia can be as high as 49.4% (81). The prevalences of periodontopathogenic bacteria in systemic vascular tissue following periodontal procedures and in atheromatous lesions without periodontal procedure in subjects with chronic periodontitis are summarized in Table 2. It can be concluded that periodontal pathogens could potentially invade the systemic vascular tissue following periodontal procedures as well as contribute to atheromatous lesions. As Koch's postulate cannot be applied in humans, the direct cause-effect of these periodontopathogenic bacteria in development of atherosclerosis still needs to be confirmed.

**Table 2:** Selected studies on bacteremia of periodontal pathogens and periodontal pathogens identified in atheromatous lesions in subjects with chronic periodontitis.

**References**	**Design**	**Technique**	**Intervention**	**Bacterial species (prevalence)**
Forner et al. (82)^a^	C-S	Lysis filtration	Chewing gum and tooth brushing	*Pg* (10), *Pi* (40%), *Fn* (40%)
Lafaurie et al. (83)^a^	C-S	Culture	SRP	*Pg* (28.5%), *Tf* (7.1%), *Fn* (11.9%)
Perez-Chaparro et al. (84)^a^	C-S	Culture	SRP	*Pg* (43.7%)
Castillo et al. (85)^a^	C-S	Nested PCR	SRP	*Pg* (31%), *Aa* (21.4)
Waghmare et al. (86)^a^	C-S	Culture	SRP	*Pg* (37.5%), *Pi* (15%), *Tf* (12.5%)
Sharmann et al. (87)^a^	RCT	Lysis centrifugation, culture	SRP and povidone iodine	*Pi* (5.2%), *Fn* (5.2%)
Marin et al. (88)^a^	C-S	Lysis centrifugation, culture, qPCR	Tooth brushing	*Fn* (33%)
Balejo et al. (79)^a^	RCT	Culture, qPCR	SRP and chlorhexidine	*Pg* (Change in levels. By culture from 113.8 to 782.4, by qPCR from 0.5 to 512.5)
Elkaim et al. (89)^b^	C-S	Hybridization	None	*Aa* (54.4%), *Pg* (72.7%),
Nakano et al. (33)^b^	C-S	Specific PCR	None	*Aa* (35%), *Pg* (20%), *Td* (20%)
Gaetti- Jardim et al. (90)^b^	C-S	Real time PCR	None	*Aa* (46.2%), *Pg* (53.8%), *Tf* (25.6%), *Pi* (59%), *Fn* (0)
Figuero et al. (91)^b^	C-S	Nested PCR	None	*Aa* (66.7%), *Pg* (78.6%), *Tf* (61.9%), *Fn* (50%)

a *Bacteremia after periodontal procedure*.

b *Periodontal pathogen in atheromatous lesion. C-S, cross sectional; SRP, scaling and root planing; RCT, randomized clinical trial; Pg, Porphyromonas gingivalis; Tf, Tannerella forsythia; Td, Treponema denticola; Pi, Prevotella intermedia; Aa, Aggregatibacter actinomycetemcomitans; Fn, Fusobacterium nucleatum; FISH, florescence in situ hybridization; qPCR, quantitative polymerase chain reaction*.

*Proof 2. Periodontal bacteria can be found in the affected tissues* There is sufficient evidence from several studies that different oral bacterial species can be identified in atheromatous lesions using DNA, RNA, antigen and passive sequencing (91–93). Analyses of samples have shown that periodontitis subjects are at high risk for development of atherosclerosis (94).

*Proof 3. Evidence of live periodontal bacteria at the affected site* Detection of live periodontopathogenic bacteria is essential to fulfill this proof. Live *P. gingivalis* and *A. a* have been isolated from atheromatous lesions by at least two studies (95, 96).

*Proof 4. In vitro evidence of invasion of affected cell types* A number of *in vitro* studies showed that periodontopathogenic bacteria can invade different types of host cells. Studies have demonstrated invasion of endothelial cells by *P. gingivalis* (97, 98) and the mechanism as well as the importance of the particular strain type have been evaluated in a further study (99).

*Proof 5. Demonstration that periodontal bacteria can promote atherosclerosis in animal models of disease* The review provided by EFP and AAP in 2012 demonstrated evidence that periodontopathogenic bacteria can induce and promote atherosclerosis (75). *P. gingivalis* has been shown to enhance atherosclerosis in murine (100), rabbit (101), and pig (102) models. Furthermore, when mice with hyperlipidemic conditions were infected orally with *P. gingivalis, T. forsythia, T. denticola* and *F. nucleatum*, viable bacteria of these species were detected in oral epithelium, aorta and atherosclerotic plaque (21, 103).

*Proof 6. In vitro and in vivo evidence that non-invasive mutants cause significantly reduced pathology (animal model)* The importance of the strain of bacterial species in respect of invasion of vascular tissue and cells has been examined. The non-invasive fimbrillinA deficient mutant of *P. gingivalis* was not found to promote atherosclerosis and resulted in less pro-inflammatory mediators than the invasive wild type strain of *P. gingivalis* (100).

*Proof 7. Fulfill modified Koch's postulate to demonstrate that a human atheroma isolate causes disease in animal models* To achieve this proof, the periodontopathogen has to be isolated from human atheroma and lead to atheroma formation in an animal model after inoculation. A strain of *P. gingivalis* has been isolated from human atherosclerotic plaque (95). Furthermore, evidence is available that inoculation of periodontopathogenic bacteria has the ability to induce atherosclerosis in animal models (21, 103, 104). However, the strains used were not obtained from human atherosclerotic plaque; therefore, this proof has not been entirely fulfilled yet.

Overall, numerous studies are available to support proofs 1 to 6, but not yet for proof 7. Nevertheless, the evidence from the first six proofs supports that periodontopathogenic bacteria are associated with atherosclerosis.

### Observational Studies

The association between PD and atherosclerosis has been intensively examined in cohort and case control studies. In general, studies on subjects with periodontitis as defined by probing pocket depth (PPD), clinical attachment loss (CAL) and alveolar bone loss have a higher prevalence of subclinical ACVD (18). Those subjects were also suggested to exhibit higher prevalence of ACVD and risk of stroke or MI (105), increased prevalence or incidence of peripheral artery disease (106, 107), and higher prevalence of arterial fibrillation (108). Taking all the evidence from observational studies (Table 3) into account, it can be concluded that the odds ratio of atherosclerotic disease is greater in patients with PD in comparison to non-PD individuals. Furthermore, The results of some interventional studies have suggested that some preventive oral hygiene measures such as regular toothbrushing and oral health interventions including self-performed oral hygiene habits (124), dental prophylaxis (103), increased self-reported visits to the dental office (123) and periodontal treatment (125, 126) can reduced the incidence of ACVD events.

**Table 3:** Summary of observational studies.

**References**	**Design**	**N**	**PD parameters**	**ACVD parameters**	**Conclusion**
Ajwani et al. (109)	Cohort	364	CPITN	Cardiovascular mortality	Periodontitis (CPITN code ≥3) with cardiovascular mortality (HR = 2.28)
Grau et al. (110)	C-C	471	AVL, CAL	Acute ischemic lesion on brain imaging	Severe periodontitis and stroke (OR = 4.34)
Sim et al. (111)	C-C	479	PPD, CAL	Stroke as a hemorrhagic or ischemic lesion using brain images taken by CT or MRI	Periodontitis and stroke (OR = 4)
Dietrich et al. (112)	Cohort	1203	AVL, PPD	MI, angina pectoris, and fatal CHD were considered as CHD events	Periodontitis and CHD (HR =2.12)
Jimenez et al. (113)	Cohort	1137	AVL, PPD	Cerebrovascular disease was defined as a cerebrovascular event consistent with stroke or transient ischemic attack	Periodontitis and cerebrovascular disease (HR= 3.52)
Xu and Lu (114)	Cohort	10849	PPD, CAL	Cardiovascular mortality	Periodontitis with cardiovascular mortality (HR = 1.64)
Hayashida et al. (115)	C-C	1053	PPD	Carotid intima media thickness, arterial stiffness (cardio-ankle vascular index)	Each 1-mm increase in mean PPD corresponded to a 0.02 mm increase in maximal carotid intima media thickness and 0.1 increase in mean cardio-ankle vascular index
Heaton et al. (116)	Cohort	1461	AVL	CHD events weredefined as MI, angina pectoris and fatal CHD	Progression of >0.5mm AVL associated IR of 5.4 and 2.5 for new and already diagnosed periodontitis subjects.
Vedin et al. (117)	Cohort	15824	Number of present teeth, BOP	Prior MI, prior coronary revascularization	Tooth loss and BoP associated with risk factors of cardiovascular disease
Ahn et al. (106)	C-S	1343	AVL, periodontitis (no, moderate, severe)	Subclinical atherosclerosis: carotid intima media thickness ≥0.754 mm, peripheral arterial disease: ankle-brachial index ≤1.0	Severe periodontitis and subclinical atherosclerosis (OR = 1.55) Severe periodontitis and peripheral arterial disease (OR = 2.03)
Gorski et al. (118)	C-C	289	PPD, BoP, CAL, tooth loss	MI according to European Society of Cardiology guideline	Periodontitis and MI (OR = 2.4).
Hansen et al. (119)	Cohort	17691	Hospital diagnosis of periodontitis	Hospital diagnosis of cardiovascular disease	Periodontitis and cardiovascular disease death (IR= 2.02)
Rydén et al. (46)	C-C	1610	AVL, PPD, BoP	First MI according to international criteria (120)	Increased risk for first myocardial infarction with periodontitis (OR = 1.49)
Bengtsson et al. (121)	C-S	499	AVL, PPD, BoP	Carotid calcification in panoramic dental radiographs	Periodontitis and carotid calcifications (OR = 1.5)
Beukers et al. (13)	Cohort	9730	CPITN	Acute cardiovascular disease	Periodontitis and acute cardiovascular disease (OR = 2.52)
Nordendahl et al. (122)	C-C	1577	AVL, PPD, BoP	First MI according to international criteria (120)	Increased risk for first MI with periodontitis in females aged <65 years (OR = 3.72)
Sen et al. (123)	C-S	17098	2017 classification (seven classes A-G) according to periodontal severity (A = periodontally healthy, G = severe periodontitis)	Subjects from atherosclerosis risk communities	Increased stroke risk with worse periodontal severity (IR = 5.03)

*C-C, case control; C-S, cross sectional; OR, odds ratio; HR, hazard ratio; IR, incident rate; PPD, probing pocket depth; CAL, clinical attachment loss; BoP, bleeding on probing; AVL, alveolar bone loss; CPITN, community periodontal index of treatment need; CHD, coronary heart disease; CT, computerized tomography; MRI, magnetic resonance imaging; MI, myocardial infarction*.

### Intervention Studies

The effect of periodontal therapy on primary prevention of ACVDs such as ischemic heart disease and cardiovascular death has not been examined to date due to methodological, financial and, most importantly, ethical considerations (76). Therefore, surrogate markers of cardiovascular events have been examined rigorously and periodontal therapy has shown significant influence on these markers as summarized in Table 4. However, evidence regarding the long-term effect of periodontal therapy on these surrogate markers is scarce. Further, the effect of periodontal therapy on clinical outcomes of cardiovascular events has not been examined yet (76).

**Table 4:** Selected studies that have demonstrated effects of periodontal therapy on cardiovascular risk markers.

**References**	**N**	**Study design**	**Cardiovascular risk markers**	**Intervention**	**Duration**	**Effect**
López et al. (120)	315	RCT	TC, HDL, and LDL and glucose, CRP, and FGN	NSPT+ amoxicillin and metronidazole	6 months	Only CRP and Fibrinogen ↓
Bokhari et al. (127)	246	RCT	CRP, FGN and white blood cells.	NSPT	2-months	All ↓
Bresolin et al. (60)	33	Prospective clinical study	CRP, TC, VLDL, HDL, TGs, FGN, IL-6, and TNF-α	NSPT	180 days	All ↓ except TNF-α
Vidal et al. (59)	26	Cohort pilot study	CRP, FGN, IL6, SBP, DBP, LVM, and PWV	NSPT	3 months 6 months	All ↓ after 6 months
Banthia et al. (128)	40	Clinical study	TLC, DLC and platelet count, BT and CT	NSPT	2 weeks	All ↓
Caúla et al. (36)	66	RCT	CRP, ESR, TC, HDL, LDL and TGs	NSPT	2 months 6 months	All ↓ except HDL ↑
Kiany and Hedayati, 2015 (129)	25	RCT	IgM aCLA, IgG aCLA	NSPT	6 weeks	All ↓
Gupta et al. (130)	150	cross-sectional	CRP	SPT	3 months	All ↓
Graziani et al. (131)	38	RCT	CRP, IL6 and TNF-α	NSPT	1 day 1 week 3 months	All ↑ after 24 hrs. but ↓ after 1week and 3 months
Houckenet al. (132)	109	Case–control and pilot intervention	Pulse-wave velocity (PWV), SBP, DBP, TC, HDL, and LDL	NSPT	3 months 6 months	PWV not changed and the others ↓
Torumtay et al. (133)	50	case–control	CRP, IL6, IL-10, TAC, TOS, FPG, HbA1c, TRG, TC, HDL, LDL, SBP and DBP	NSPT	3 months 6 months	All ↓ except HbA1c, SBP, DBP unchanged after 6 months.
Siddeshappa et al. (134)	30	clinical trial	TLC, platelet count	NSPT	1 week and 2 weeks	All ↓
Arvanitidis et al. (135)	25	clinical trial	Binding of PAC-1, P-selectin and CD63, TLC and platelet count	NSPT	3 months	All ↓
Zhou et al. (136)	107	RCT	SBP, DBP, EM, CRP, IL-6	intensive periodontal treatment	1 months 3 months 6 months	SBP ↓ but DBP, EM, and CRP ↓ after 3 and 6 months but IL-6 ↓ only after 6 months
de Souza et al. (137)	44	RCT	CRP	NSPT	60 days	All ↓
Jockel-Schneider et al. (138)	55	RCT	PWV, PPao, RRsys, Aix, and MAP	NSPT + amoxicillin (500 mg) and metronidazole (400 mg),	12 months	PWV ↓, PPao ↑ RRsys and MAP not changed
Saffi et al. (139)	69	RCT	FMD, sVCAM-1, sICAM-1, and P selectin	NSPT	3 months	All ↓ except FMD
Morozumi et al. (140)	31	RCT	CRP, IFN-γ, IL-5, IL-6, IL-12, TNF-α	NSPT	1 day 6 weeks	All ↑ after 1 day After 6 weeks: CRP, IFN-γ and IL-6 ↓ IL-5, IL-12, TNF-α ↑
Moeintaghavi et al. (141)	30	RCT	TC, LDL, HDL, TGs, CRP), and FBS.	SPT and NSPT	3 months	All ↓ except HDL

*RCT, randomized clinical trial NSPT, non-surgical periodontal therapy; SPT, surgical periodontal therapy; TC, total cholesterol; HDL, high-density lipoprotein; TGs, triglycerides; LDL, low-density lipoprotein; FGN, fibrinogen; CRP, C-reactive protein; IL, interleukin; TNF, tumor necrosis factor; ESR, erythrocyte sedimentation rate; LVM, left ventricular mass; TAC, total antioxidant capacity; TOS, Total oxidant status; TLC, Total leucocyte count; DLC, differential leucocyte count; BT, bleeding time; CT, clotting time; EM, Endothelial Microparticles; FMD, flow-mediated dilation; PWV, pulse wave velocity; Aix, augmentation index; PPao, central pulse pressure; RRsys, peripheral systolic pressure; MAP, Mean arterial pressure; DBP, diastolic blood pressure; SBP, systolic blood pressure; FPG, fasting plasma glucose; HbA1c, glycated hemoglobin; FBS, Fasting Blood sugar. ↓ mean decrease, ↑ mean increase*.

The majority of researches relating periodontal treatment to ACVD have focused on assessing and quantifying systemic inflammatory biomarkers and endothelial function, as these atherosclerotic risk factors allow the estimation of treatment outcomes over a shorter period. Previous studies have shown that intensive periodontal treatment temporarily increases the blood levels of inflammatory markers, and worsens endothelial function, possibly through the release of bacteria or inflammatory cytokines into the blood stream. However, after several weeks, local and systemic inflammatory markers as well as PD parameters are lower, with considerably better endothelial function than before treatment (142–145). Moreover, carotid intimal-medial thickness is decreased 6 months after periodontal treatment (146). Several randomized controlled clinical trials, case control studies and systematic reviews with meta-analyses have been published in the last decade (Table 4) and they support the notion that periodontal treatment has an effect on cardiovascular events by reducing many cardiovascular risk factors.

Future intervention researches are needed to further clarify the relationship between PD and ACVD, particularly in terms of the biological impacts of PD on the atherogenic cascade by influencing the vascular endothelium. At the same time, there is still a need for more long-term interventional studies, preferably using more homogeneous methodologies for evaluating ACVD events, to determine whether the stated advantages of periodontal treatment translate into a reduction of ACVD occurrence.

## Effects of Statins as Medication for ACVD on PD

Among the different medications used for treatment and prevention of ACVD, statins have demonstrated therapeutic potential in treating PD, which was further confirmed when used via a local delivery system (147, 148). Statins are inhibitors of 3-hydroxy-methylglutaryl coenzyme A reductase (HMG-CoA reductase). These medications have different ring structures and they are known to reduce the level of LDL and cholesterol in the blood for the prevention of ACVD (149, 150). Apart from their main action of lowering lipid levels, statins have several pleiotropic effects including anti-inflammatory, antioxidative, antibacterial and immunoregulatory functions (151, 152).

The anti-inflammatory effect of statins is due to their capability to inhibit pro-inflammatory cytokines and upregulate anti-inflammatory and/or proresolution molecules. This effect is primarily attributed to the activation of extracellular signal-regulated protein kinases (ERK), mitogen activated protein kinase (MAPK), protein kinase (PI3-Akt) signaling pathway, and suppression of NF-κB activation pathways. Furthermore, statins are able to modulate host response to bacterial challenges; thereby preventing inflammation-mediated bone resorption and stimulating new bone formation (153). Local delivery of statins using experimental animal models contributed in preventing alveolar bone resorption as a result of their anti-inflammatory, anti-microbial and bone remodeling properties besides their metalloproteinases inhibitory effect (154).

A 5-year population based competitive follow-up study investigated the effect of systemically administrated statins on the tooth loss rate as compared to participants not on statin medications. The study reported a reduction in the incidence of tooth loss in patients on statin therapy compared to controls (155). In addition, significant improvement in clinical signs of periodontal inflammation was recorded compared to those not taking statins (148).

**Figure 2:**
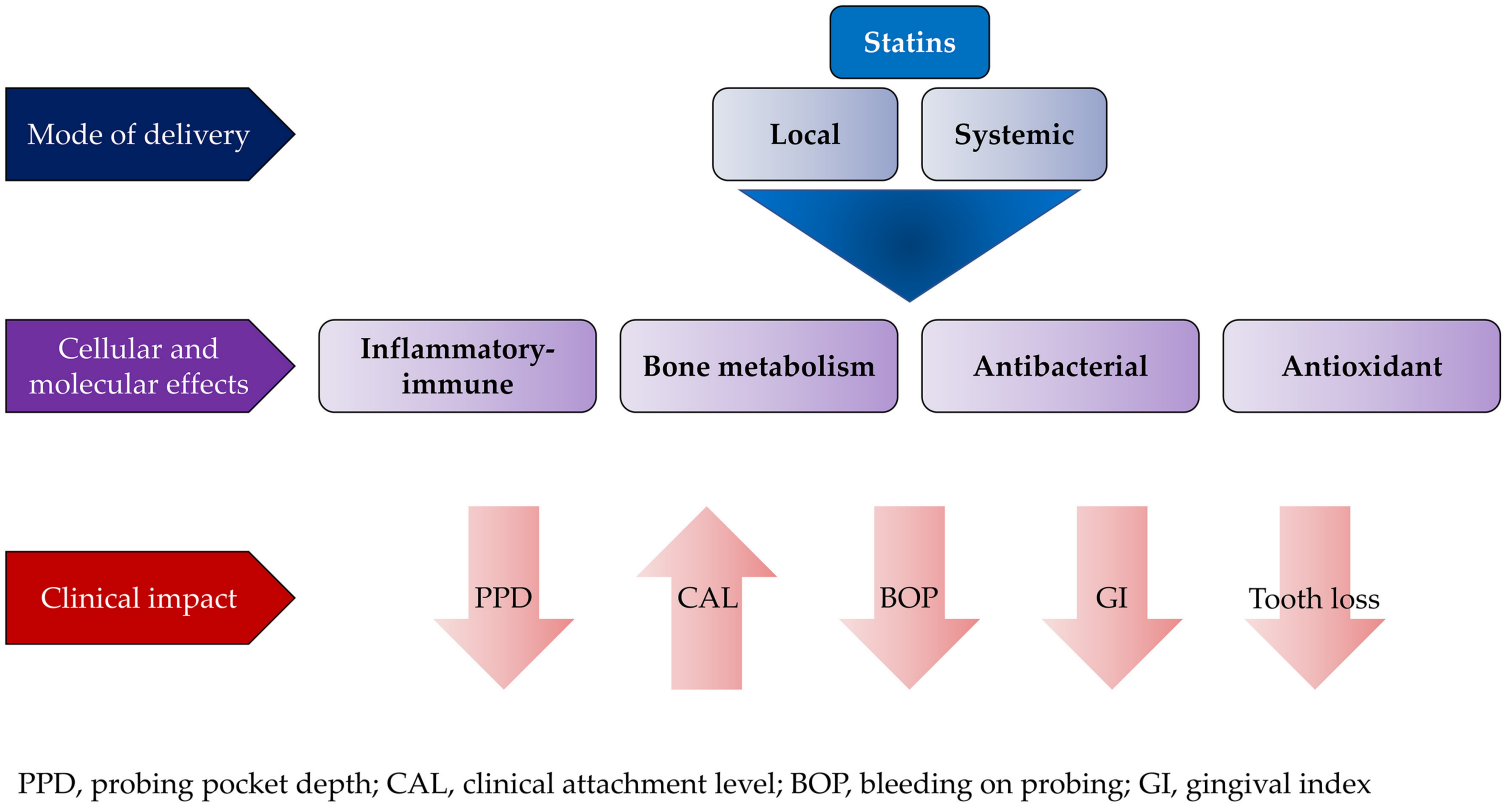
Effect of local and systemic use of statins on the outcome of conventional periodontal treatment.

The effect of statins on periodontitis in otherwise healthy patients was assessed in a review by Petit et al. (153). The primary outcomes included CAL, PPD, and gingival index as well as level of inflammatory biomarkers in serum and gingival crevicular fluid. For clinical parameters, contradictory results were reported on using statins as local adjunct to non-surgical therapy. However, most of these studies consistently reported significant reduction in proinflammatory mediators such as IL-8 and IL-6 associated with upregulation of anti-inflammatory cytokines such as IL-10 (156–158). To the contrary, local application of statins with different periodontal surgeries resulted in significant improvement in PPD, CAL, and bony defects (159–162). The systemic impact of statins on the outcomes of non-surgical periodontal treatment have not been fully elucidated. There are discrepancies in the results of the available studies that are mainly due to variations in the follow-up period and/or their design (153, 163). Cellular and molecular effects of statins on periodontal tissues and their clinical impact are illustrated in Figure 2.

In general, local application of statins was found to achieve better treatment outcome than systemic application when used as an adjunct to periodontal therapies. Despite the promising results of statins, their effects on different aspects of soft and hard tissue healing need further exploration, especially on wound healing and regeneration.

## Clinical Significance of the Link Between PD and ACVD for Dental Practitioners and Cardiologists

As detailed previously, a substantial body of evidence supports the relationship between PD and ACVD. Although many studies have reported that periodontal therapy significantly increases surrogate markers of ACVD within a short time, followed by improvement in systemic inflammation and endothelial function (76, 164), invasive dental procedures including periodontal treatment have not been associated with increased risk of MI (165). Furthermore, hemoglobin A1c (Hb A1c) has been found to decrease after periodontal therapy, which is of clinical relevance (143).

Dental practitioners have to be aware of the association between these two diseases. Patients with severe periodontitis should be advised to see a physician to check for signs of ACVD. Those patients should be informed that PD is associated with increased risk of cardiovascular complications and therefore their periodontal condition requires treatment. Furthermore, subjects with ACVD have to adhere to proper oral hygiene measures and regular check-ups with a dental practitioner (18, 76).

Although there is lack in evidence of a direct cause-effect relationship between PD and ACVD, evidence from published studies have confirmed the reduction in the systemic burden of inflammation following periodontal therapy. Thus, cardiologists should notify patients with atherosclerosis about the importance of good oral and dental health. Patients should be advised of the need to have regular home and professional dental care. Furthermore, the physician can recommend referral to a dentist or periodontist for oral and periodontal examination, assessment and treatment when necessary (76). Cooperation between the dentist and the cardiologist is of paramount importance for patients on anticoagulant/antiplatelet medication prior to any oral or periodontal surgeries to avoid any complications such as excessive bleeding and ischemic events.

## Conclusions and Future Research

Evidence from the studies detailed in this review supports the notion that there is a link between PD and ACVD. These two diseases share several systemic inflammatory mechanisms including increases in levels of inflammatory mediators, lipids, and hemostatic and thrombotic factors. Furthermore, they share several risk factors such as smoking and genetics. However, the extent of the impact of PD on the initiation and progression of ACVD is not clear yet and needs to be further examined. Microbiological studies have shown that periodontal pathogens can cause bacteremia and invasion of distant tissues. Evidence from epidemiological studies shows that the odds ratio of atherosclerotic disease is greater in patients with PD in comparison to non-PD individuals. Interventional studies could not examine the effect of periodontal therapy on primary prevention of ACVD such as ischemic heart disease and cardiovascular death due to methodological, financial and, most importantly, ethical considerations. Therefore, surrogate markers of cardiovascular events have been examined rigorously and periodontal therapy has shown significant influence on these markers in the short term. On the other hand, amongst medications used for treatment and prevention of ACVD, statins have shown positive impact on periodontal treatment outcome. Several mechanisms have been proposed regarding the effect of statins on PD outcome, but again this needs to be further investigated. Therefore, it is too early to mark PD as a causal factor with direct relation to the etiology and incidence of ACVD. Further studies should be conducted *in vivo* and *in vitro* to determine the cause-effect relationship between PD and ACVD, besides longitudinal studies and with longer follow up are advised to provide solid confirmation to support this relationship and clarify the link between PD and ACVD.

## Author Contributions

FZ: conceptualization and first draft of sections Introduction, Biomarkers Shared by Periodontal and Atherosclerotic Cardiovascular Diseases, and Clinical Significance of the Link Between PD and ACVD for Dental Practitioners and Cardiologists. SG: conceptualization and first draft of sections Effects of Periodontal Disease on the Incidence of Atherosclerotic Cardiovascular Diseases and Conclusions and Future Research. AA: drawing the figures and first draft of sections Pathogenesis of Atherosclerotic Cardiovascular Diseases Including the Role of Periodontal Disease, Confounders Between Periodontal and Atherosclerotic Cardiovascular Diseases, and Biomarkers Shared by Periodontal and Atherosclerotic Cardiovascular Diseases. AS: first draft of sections Effects of Periodontal Disease on the Incidence of Atherosclerotic Cardiovascular Diseases and Effects of Statins as Medication for ACVD on PD. JY, SG, and AA: critically reviewed the manuscript and final version editing. All authors have read and agreed to the published version of the manuscript.

## Conflict of Interest

The authors declare that the research was conducted in the absence of any commercial or financial relationships that could be construed as a potential conflict of interest.
